# Influence of different personal protective equipment on children’s anxiety in dental office: a randomized controlled trial

**DOI:** 10.1186/s12903-022-02442-5

**Published:** 2022-09-22

**Authors:** Maha Moussa Azab

**Affiliations:** 1 Department of Pediatric Dentistry, Faculty of Dentistry, Fayoum University, Fayoum, Egypt; 2 Discipline of Pediatric Dentistry, School of Dentistry, Newgiza University NGU, Giza, Egypt

**Keywords:** Dental anxiety, PPE, Reusable respirators, Dentist’s attire

## Abstract

**Background:**

A change in how a dentist looks may affect the child’s anxiety in the dental office. This study compared the effect of conventional facial PPE versus extra PPE as reusable respirators; on the preoperative child’s anxiety in the dental office.

**Methods:**

Fifty two children were randomly allocated into 4 groups, (1) goggles + surgical mask, and (2) face shield + surgical mask versus (3) half-face respirator and (4) full-face respirator. Each child was communicated with and clinically examined by a dentist wearing the assigned PPE, and then his anxiety was assessed using CFSS-DS. Shapiro–Wilk’s test was used to analyse normality. Kruskal–Wallis test followed by Dunn’s post hoc test with Bonferroni correction test, were used to analyse non-parametric anxiety score data. Correlations between different factors and anxiety scores were analysed using Spearman’s rank-order correlation coefficient.

**Results:**

There were no statistically significant differences in the number of anxious children in each group, boys had significantly higher scores than girls (*p* < 0.001) For the “Goggles and surgical mask” group and “overall”. There was no significant correlation between age and anxiety scores. Children who didn’t have a previous dental visit had statistically significant higher scores than children with previous experience for “Goggles and surgical mask”, “Face shield and surgical mask “groups and “overall”.

**Conclusions:**

Half-face and full-face respirators have not affected the child’s preoperative anxiety in the dental office when compared to the conventionally used PPE. Overall, there is an association between gender and previous dental visits, and dental anxiety, however; there is no correlation between child’s age and dental anxiety. Dentists dealing with children should feel free to use reusable respirators, without the risk of affecting children’s anxiety in the dental office.

*Trial Registration* This study was registered on www.clinicaltrials.gov, ID: NCT05371561 on 12/05/2022.

## Background

During the last couple of years, the rise of the COVID-19 pandemic has set new norms for life in general and more specifically for the life of health care workers. Dental practitioners are at higher risk to be exposed to COVID-19 than other health-related professions [1]. As a result of the COVID-19 pandemic, various organizations in different countries have set updated guidelines for treating patients in dental settings, some of these recommendations addressed the dentist’s personal protective equipment (PPE), and recommended the use of extra protection such as reusable respirators, especially when performing aerosol-generating procedures [2].

Conventional surgical masks are designed to protect from large respiratory droplets and might be considered enough during conventional, non-aerosol generating procedures, on the other hand, N95 respirators give better protection including protection from aerosols; however, there is the drawback of high cost which makes them not very practical to use routinely. For many, the use of reusable respirators was a more practical solution that deliver the required protection, in his review Sozkes et al. concluded that reusable respirators are more fitting, more stable and less prone to leakage compared to surgical masks and N95 respirators [3, 4].

The change in the way the health care worker looks has an impact on patients’ attitudes in health care facilities, Shulman et al. [5] found that adults -in military and civilian hospitals- were satisfied with, and preferred dentists wearing masks and protective glasses for better infection control practices. However, when dealing with children, the effect of such change in the dentists’ PPE and subsequently how they look, should be investigated to test its effect on the child’s anxiety in the dental office.

Several studies have studied the effect of dentists’ attire on the child’s anxiety and behaviour in dental settings; Kuscu et al. [6] found that 45.6% of 9-to 14-year-old children preferred formal attire; however, anxious children preferred “child-friendly” attire, Babaji et al. [7] found that children of different age groups have different preferences regarding their dentists’ outfit. Asokan et al. [8] studied dentists’ PPE and found that anxious 9–12 year-old children preferred coloured attire and dentists with protective wear. Recently, Berwick et al. [9] studied the effect of extra PPE forced by the COVID-19 pandemic on preoperative anxiety of children preparing for surgeries in the anaesthetic room.

Being paediatric dentists where the child’s behaviour is a crucial concern in our work, and where it is our responsibility to instill a positive attitude towards dental visits, we should study the effect of each practice that may affect children’s anxiety and subsequently their attitude and behaviour. In the time of a pandemic as COVID-19 or when dealing with any child with a serious respiratory infection, will the use of reusable respirators that provide extra protection affect the child’s anxiety in a dental setting? To my knowledge this is the first study to address the effect of the recommended extra PPE on children’s preoperative fear and anxiety in the dental office; so, the current study aims to compare the effect of conventional facial PPE as (1) goggles + surgical mask, and (2) face shield + surgical mask versus (3) half-face reusable respirator and (4) full-face reusable respirator; on preoperative child’s anxiety in the dental office.

## Methods

This randomized controlled trial was reported according to the CONSORT (Consolidated Standards of Reporting Trials) statement [10]. The study was approved by (Fayoum University Supreme Committee for Scientific Research Ethics (FU-SCSRE). The study was registered on www.clinicaltrials.gov, with the ID: NCT05371561 on 12/05/2022.

### Sample size

According to Asokan et al. [8], the percentage of anxious children who preferred dentists with protective wear was nearly 70%. Using the G*power program (version 3.1.9.4) for sample size determination, A total sample size (n = 50, ≈13 patients in each group) will be sufficient to detect an effect size of 0.4, with an actual power (1-*β* error) of 0.8 (80%) and a significance level (*α* error) 0.05 (5%) for the two-sided hypothesis test.

### Study setting

Outpatient clinic, Faculty Dentistry, Fayoum University-Egypt.

### Eligibility criteria

Healthy 6–10 year-old patients who do not need emergency treatment. Patients who have systemic, mental or psychological conditions as indicated by their medical history, were excluded from the study.

Before enrolling children in the study, written consent was taken from each legal guardian, and children were asked if they agree to answer several questions.

### Randomization and allocation

**Fig. 1:**
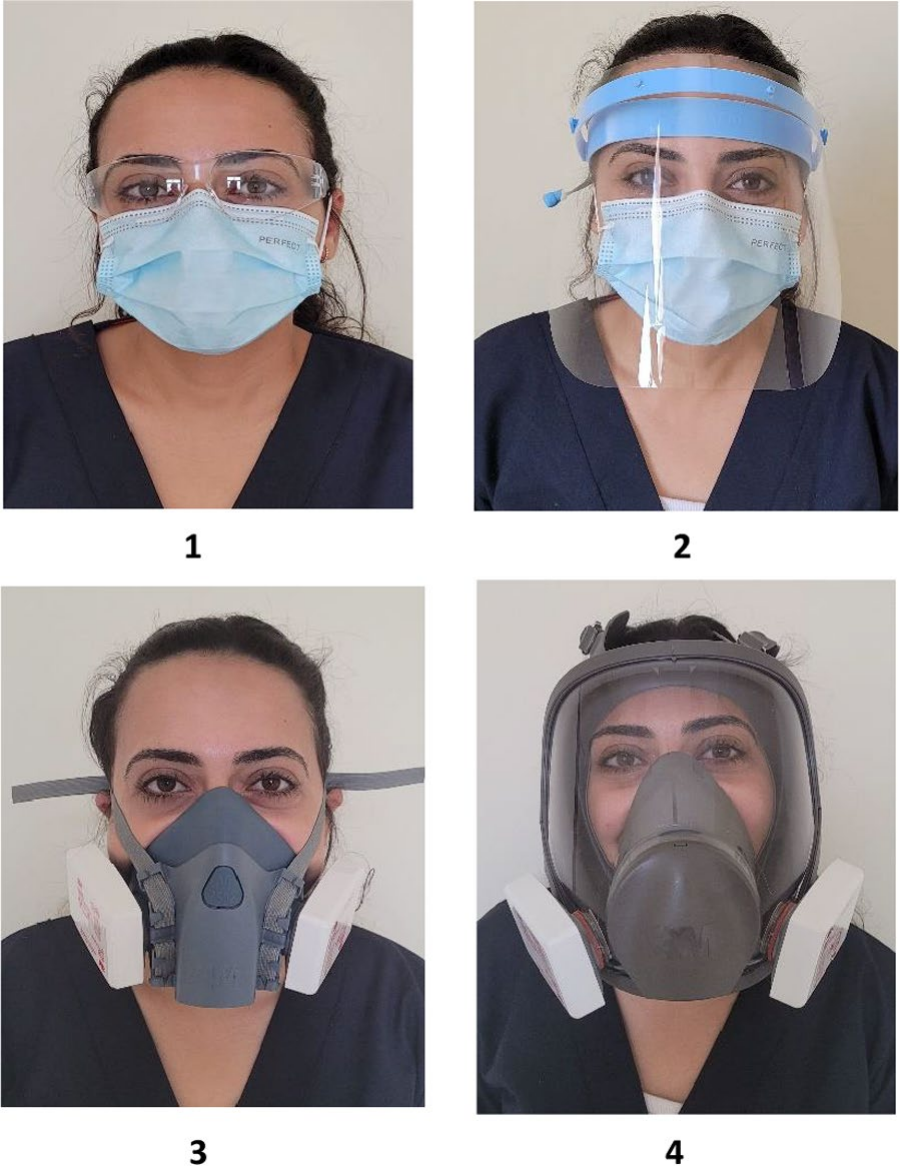
PPEs in each study group, group **1**: Goggles + Surgical mask, group **2**: Face shield + Surgical mask, group **3**: Half-face reusable respirator + Filter, and group **4**: Full-face reusable respirator + Filter

Each child was randomly assigned to be examined by a dentist using one of the under-study PPEs shown in Table 1 and Fig. 1. This was done by choosing an envelope from a batch of opaque, sealed envelopes containing the specific PPE (study group). This procedure was done by an administrator who is not otherwise involved in the study. The administrator will draw an envelope for each child included in the study, and deliver it to the examiner dentist, to put on the selected PPE just before the child gets into the examination room.

**Table 1:** PPE used for each study group

Group	PPE-study group	Brand name	Manufacturer
1	Goggles + surgical mask	3 M™ SecureFit™ 100 series safety glasses (clear lens)	3 M, Taiwan
		Evony surgical mask	Hayat Egyptian hygienic products, Egypt
2	Face shield + surgical mask	Cotisen plastic disposable full cover protective face shield	Huanghua Promisee Dental Co., Ltd. China
		Evony surgical mask	Hayat Egyptian hygienic products, Egypt
3	Half-face reusable respirator + filter	3 M™ half facepiece Reusable Respirator 7502	3 M USA
		3 M™ particulate Filter P3 R, 6035	3 M Canada
4	Full-face reusable respirator + filter	3 M™ full facepiece Reusable Respirator 6800	3 M Poland
		3 M™ particulate filter P3 R, 6035	3 M Canada

### Blinding

It was inapplicable to blind the participants, legal guardians, and the dentist who carried out the examination. The accessor and statistician were blinded.

### Procedure

**Fig. 2:**
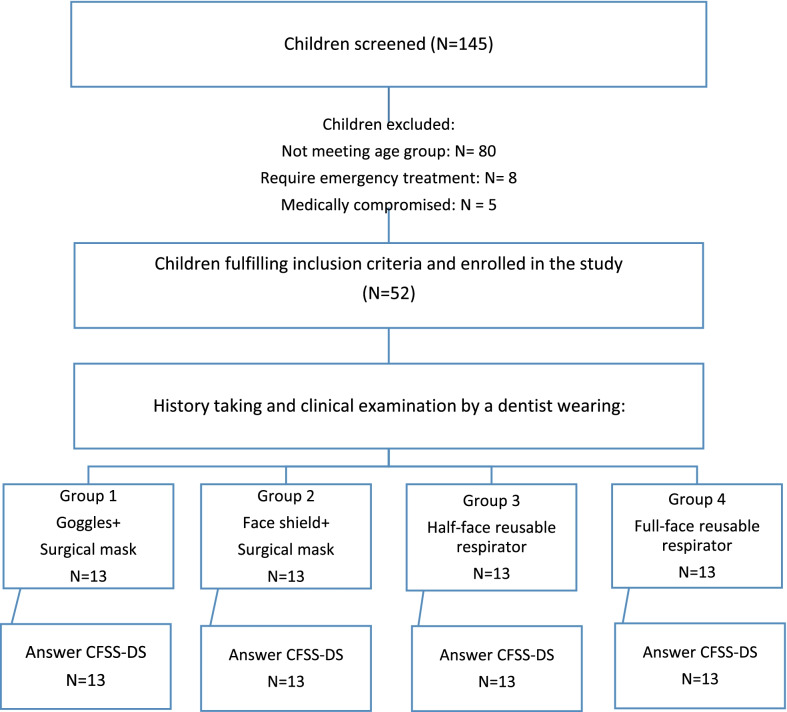
Study flowchart

Each patient meeting the inclusion criteria and assigned to a specific study group entered the clinic for the dental examination. The examining dentist wearing the selected PPE, communicated with the patient and parent, took personal, previous and current medical and dental history, and carried out a simple examination not involving provoking pain, the sight of sharp instruments, or investigations such as radiographs. The process was planned to take from three to five minutes. Then, the child will be escorted to another room to meet a blinded assessor, who assesses the child’s anxiety using the Arabic version of CFSS-DS. The study flow chart is presented in Fig. 2.

The original English version of the CFSS-DS was developed by Cuthbert et al. 1982 and was used in previous studies to measure dental fear and anxiety in children [11–13], the Arabic version was used in this study to facilitate communication with Arabic-speaking children [14]. The Arabic version of the CFSS-DS has high reliability, good criterion validity, and moderate construct validity [15]. In the current study, children answered the questions themselves rather than their parents, as parents tend to overestimate their children’s fear [9].

The CFSS-DS consists of 15 items, revolving around dental settings and procedures, children answered each item by choosing a rate from a 5-point Likert scale that scores as Not afraid: (1), a little afraid: (2), fairly afraid: (3), quite afraid: (4), and very afraid: (5), the total score should range from 15 to 75, and a score of 32 or more is considered anxious [11].

### Statistical analysis

Categorical data were presented as frequency and percentage values and were analysed using the chi-square test. Numerical data were presented as mean and standard deviation (SD) values and were analysed for normality using Shapiro–Wilk’s test. Anxiety score data were non-parametric and were analysed using the Kruskal–Wallis test followed by Dunn’s post hoc test with Bonferroni correction. Correlations between different factors and anxiety scores were analysed using Spearman’s rank-order correlation coefficient. The significance level was set at *p* < 0.05 within all tests. Statistical analysis was performed with R statistical analysis software version 4.1.3 for Windows [16].

## Results

52 children, 25 girls and 27 boys, with mean age of (7.71 ± 1.36) years were enrolled in the study, of the enrolled children 25 were on their first dental visit, while 27 have visited a dental clinic before.

For each item of CFSS-DS and total anxiety scores, there were no statistically significant differences between study groups (*p* > 0.05). Intergroup comparisons for each item and total scores were presented in Tables 2 and 3.

**Table 2:** Intergroup comparisons for anxiety score

Question are you afraid of…	Score (mean ± SD)				Statistic	*p*-value
	Goggles and surgical mask	Face shield and surgical mask	Half-face reusable respirator	Full-face reusable respirator		
Dentists	1.77 ± 1.54^A^	1.54 ± 1.33^A^	1.38 ± 0.87^A^	2.23 ± 1.74^A^	2.15	0.542
Doctors	1.31 ± 0.75^A^	1.23 ± 0.83^A^	1.23 ± 0.60^A^	1.23 ± 0.83^A^	0.53	0.912
Injections shots	2.31 ± 1.75^A^	2.46 ± 1.76^A^	3.31 ± 1.38^A^	2.85 ± 2.08^A^	2.81	0.422
Having somebody examine your mouth	1.46 ± 1.20^A^	1.31 ± 0.75^A^	1.85 ± 1.21^A^	1.38 ± 0.96^A^	3.98	0.264
Having to open your mouth	1.54 ± 1.33^A^	1.15 ± 0.55^A^	1.54 ± 1.20^A^	1.62 ± 1.26^A^	1.41	0.702
Having a stranger touch you	1.54 ± 1.33^A^	1.23 ± 0.83^A^	1.23 ± 0.60^A^	1.69 ± 1.11^A^	2.26	0.521
Having somebody look at you	1.92 ± 1.50^A^	1.00 ± 0.00^A^	1.46 ± 1.20^A^	1.54 ± 1.05^A^	4.67	0.198
The dentist drilling	2.00 ± 1.63^A^	2.92 ± 1.93^A^	2.62 ± 1.89^A^	1.85 ± 1.34^A^	3.46	0.326
The sight of the dentist drilling	2.00 ± 1.63^A^	2.31 ± 1.55^A^	2.31 ± 1.80^A^	1.85 ± 1.34^A^	0.85	0.837
The noise of the dentist drilling	2.00 ± 1.63^A^	2.00 ± 1.58^A^	2.08 ± 1.75^A^	1.85 ± 1.34^A^	0.10	0.992
Having somebody put instruments in your mouth	1.92 ± 1.50^A^	1.54 ± 1.33^A^	1.69 ± 1.11^A^	1.46 ± 1.13^A^	1.26	0.739
Choking	1.92 ± 1.50^A^	2.23 ± 1.64^A^	2.15 ± 1.34^A^	2.15 ± 1.57^A^	0.33	0.954
Having to go to the hospital	1.69 ± 1.38^A^	1.62 ± 1.19^A^	1.46 ± 1.20^A^	1.69 ± 1.11^A^	0.59	0.899
People in white uniforms	1.62 ± 1.50^A^	1.38 ± 0.96^A^	1.00 ± 0.00^A^	1.54 ± 1.05^A^	2.92	0.404
Having the dentist clean your teeth	1.69 ± 1.38^A^	1.69 ± 1.18^A^	1.31 ± 0.75^A^	1.62 ± 1.19^A^	0.94	0.815
Overall	1.78 ± 1.42^A^	1.71 ± 1.33^A^	1.77 ± 1.33^A^	1.77 ± 1.32^A^	1.05	0.789

**Table 3:** Intergroup comparison for child’s anxiety

Anxious		Goggles and surgical mask	Face shield and surgical mask	Half-face reusable respirator	Full-face reusable respirator	Statistic	*p*-value
No	n	10	9	9	10	0.390	0.942
	%	76.9%	69.2%	69.2%	76.9%		
Yes	n	3	4	4	3		
	%	23.1%	30.8%	30.8%	23.1%		

For the “Goggles and surgical mask” group and “overall”, boys had significantly higher scores than girls (*p* < 0.001). While for other groups, there was no significant association between gender and anxiety score (*p* > 0.05). Associations between anxiety and gender are presented in Table 4.

**Table 4:** Association between anxiety and gender

Group	Score (mean ± SD)		Statistic	*p*-value
	Male	Female		
Goggles and surgical mask	2.36 ± 1.69	1.10 ± 0.48	6553.00	< 0.001*
Face shield and surgical mask	1.56 ± 1.32	1.75 ± 1.34	3663.00	0.244
Half-face reusable respirator	1.87 ± 1.40	1.63 ± 1.21	4803.50	0.327
Full-face reusable respirator	1.90 ± 1.42	1.48 ± 1.02	4567.50	0.069
Overall	1.97 ± 1.49	1.53 ± 1.13	86,217.00	< 0.001*

*; significant (*p* ≤ 0.05)

Subgroup analysis was done for 2 age groups 6–8 and 8–10, for all study groups and overall, there was no significant correlation between age and anxiety scores (*p* > 0.05). Correlations between anxiety and age are presented in Table 5.

**Table 5:** Correlation between anxiety and age subgroups

Group	Score (mean ± SD)		Statistic	*p*-value
	6–8 years	8–10 years		
Goggles and surgical mask	1.67 ± 1.20	1.83 ± 1.51	4153.50	0.708
Face shield and surgical mask	1.75 ± 1.39	1.66 ± 1.26	4807.00	0.780
Half-face reusable respirator	1.75 ± 1.29	1.83 ± 1.43	4162.50	0.703
Full-face reusable respirator	1.77 ± 1.33	1.77 ± 1.32	4527.00	0.930
Overall	1.74 ± 1.31	1.77 ± 1.40	75,924.00	0.894

For “Goggles and surgical mask”, “Face shield and surgical mask “groups and “overall”, children who didn’t have a previous dental visit had statistically significant higher scores than children with previous experience (*p* < 0.05). For other groups, there was no significant association between dental experience and anxiety scores (*p* > 0.05). Associations between anxiety and dental experience are presented in Table 6.

**Table 6:** Association between anxiety and dental visit

Group	Score (mean ± SD)		Statistic	*p*-value
	First dental visit	Not first dental visit		
Goggles and surgical mask	2.28 ± 1.70	1.47 ± 1.12	5597.50	< 0.001*
Face shield and surgical mask	1.93 ± 1.50	1.51 ± 1.14	5332.00	0.038*
Half-face reusable respirator	1.85 ± 1.31	1.65 ± 1.36	4955.50	0.141
Full-face reusable respirator	1.87 ± 1.35	1.69 ± 1.30	5052.00	0.288
Overall	1.96 ± 1.45	1.57 ± 1.22	86,182.50	< 0.001*

*; significant (*p* ≤ 0.05)

## Discussion

Dental fear and anxiety in the dental office are very common and have been proven to be a barrier to dental care for children [17]. Authors have put some effort to study how the dentist’s attire would affect the child’s anxiety in the dental office [6–8, 18].

This randomized controlled trial was carried out to compare and test the effect of different PPE on children’s anxiety in dental settings, taking into consideration the recent recommendations introduced due to the COVID-19 pandemic.

In this study, we used the commonly used PPE as goggles and face shields (groups 1 and 2) as comparators to the “new norms” in some dental settings’ half-face and full-face reusable respirators. These elastomeric respirators offer higher assigned protection factors (APFs) than conventional disposable masks, partially due to the seal developed by the elastomeric mask, and partially due to the used filter/cartridge. The reusability of these elastomeric respirators was also an advantage during the times when PPE was short [19].

The results of our study showed no statistically significant difference in preoperative anxiety between all study groups, for each item and for total CFSS-DS scores, this came in agreement with Berwick et al., who found that the use of full PPE does not cause anxiety for children in a surgical setting their findings were reported by children and confirmed by their reaction to anaesthesia induction, Berwick et al. [9] study took part during the time of COVID-19 pandemic as well. These results may be justified by the fact that children at this age are aware of the changes happening during the COVID-19 times, it is now normal to see people wearing masks all the time; so, they easily accept the change in the appearance of their health care workers.

The examiner dentist reported that she did not feel discomfort or anxiety from the children’s side during the examination; however, she reported that the half-face and full-face reusable respirators did cause some change in her voice which could affect her communication with the children.

Although not the main outcome of the study, data were analysed to study the associations and correlations of dental anxiety with other factors such as gender, age and whether the child have to the dentist before. In the current study, boys were statistically significant more anxious than girls for the “Goggles and surgical mask” group and “overall”, but other study groups showed no correlation between gender and anxiety. Previously, Rank et al. [20] studied the motivational influence of awards at the end of a dental visit, in their study, girls were less anxious than boys after receiving awards in a previous visit. The study by Mendoza-Mendoza et al. [21] found no relation between anxiety scores and gender. Disagreeing with the findings in this study, Alshoraim et al. [22] Found that 12–15 year-old girls were statistically significant more fearful than boys, in Dahal et al. study, > 50% of 6–15 years-old girls had high fear in comparison to 34.5% of boys as assessed by their responses to the CFSS-DS [12], and Majstorovic et al. [13] evaluated the relationship between dental anxiety and some child and parental characteristics; they assessed dental anxiety using CFSS-DS and found that girls were more anxious than boys (32.5 vs. 26.3, *p* = 0.003).

In the current study, there was no correlation between age and anxiety score, this may be due to the small age range for the study population; however, this came in disagreement with Mendoza-Mendoza et al. [21] who found higher anxiety scores for 4 and 5 year-old children in their study, and concluded a negative correlation between age and anxiety scores.

For “Goggles and surgical mask”, “Face shield and surgical mask “groups and “overall”, children were statistically significant more anxious when this was their first dental visit, this came in agreement with Alshoraim et al. [22] who found that children are more fearful on their first dental visit. In Mendoza-Mendoza et al. [21] study there was a moderate positive correlation between elevated anxiety scores and the number of previous dental visits. For the intervention groups in our study “Half-face reusable respirator + filter” and “Full-face reusable respirator + filter”, there was no correlation between dental experience and anxiety score, this was the case with a previous study by Rojas et al. [23] who found no association between the previous dental visits and the anxiety level of 6 year-old children.

The current study is the first study to address the use of extra PPE in dental settings and its effect on a child’s preoperative anxiety; however, more trials involving a preventive or a treatment dental procedure may be more conclusive. Also, a longer procedure would emphasize the effect of altered voice and difficulty in communication reported by the examiner dentist. Dentists can feel confident that the protection delivered by reusable respirators, does not come with the price of increased anxiety in the dental office, children can generally be more anxious on their first dental visit, that is why dentists should do their best to make the first dental visit friendly, short and non-invasive, where the primary concern is communication with the child rather than an actual dental procedure or staying safe from infections.

## Conclusions

From the results of the current study, one can conclude that half-face and full-face respirators have not affected the child’s preoperative anxiety in the dental office when compared to the conventionally used PPE such as (goggles + surgical mask) and (face shield + surgical mask). Overall, there is an association between gender and previous dental visits, and dental anxiety, however; there is no correlation between a child’s age and dental anxiety.

## Data Availability

The datasets generated and analysed during the current study are not publicly available due to ethical and privacy considerations but are available from the corresponding author on reasonable request.

## Abbreviations

PPE: Personal protective equipment
CFSS-DS: Children's fear survey schedule-dental subscale

## References

[1] Singhal S, Warren C, Hobin E, Smith B (2021). How often are dental care workers exposed to occupational characteristics that put them at higher risk of exposure and transmission of COVID-19?. Comp Anal Can Dent Assoc.

[2] Kumar G, Gugnani N, Rabea D, Odeh R, Rehman F, Mabrouk R (2021). Severe acute respiratory syndrome coronavirus 2 and dentistry: a summative review of guidelines issued by national health authorities. J Indian Soc Pedod Prev Dent.

[3] Centres for Disease Control and Prevention. Respirator trusted-source information the national personal protective technology laboratory; 2014. http://www.cdc.gov/niosh/npptl/topics/respirators/disp_part/respsource3healthcare.html. Accessed 12 Aug 2022.

[4] Sozkes S, Sozkes S (2021). COVID-19 and respiratory protection for healthcare providers. Int J Occup Med Environ Health.

[5] Shulman ER, Brehm WT (2001). Dental clinical attire and infection-control procedures patients' attitudes. J Am Dent Assoc.

[6] Kuscu OO, Caglar E, Kayabasoglu N, Sandalli N (2009). Short communication: preferences of dentist's attire in a group of Istanbul school children related with dental anxiety. Eur Arch Paediatr Dent.

[7] Babaji P, Chauhan P, Churasia VR, Kaur T, Singh S, Augustine M (2018). A cross-sectional evaluation of children preference for dentist attire and syringe type in reduction of dental anxiety. Dent Res J.

[8] Asokan A, Kambalimath HV, Patil RU, Maran S, Bharath KP (2016). A survey of the dentist attire and gender preferences in dentally anxious children. J Indian Soc Pedod Prev Dent.

[9] Berwick C, Benison E, Masters J, Robinson D, Tan J, Martin R, Okonkwo I (2021). Effect of personal protective equipment on perioperative anxiety in children and young people. Br J Anaesth.

[10] Moher D, Hopewell S, Schulz KF (2010). CONSORT 2010 explanation and elaboration: updated guidelines for reporting parallel group randomised trials. BMJ.

[11] Cuthbert MI, Melamed BG (1982). A screening device: children at risk for dental fears and management problems. ASDC J Dent Child.

[12] Dahal S, Shrestha A, Bhagat T (2020). Prevalence of dental fear among 6–15 years old school children. JNMA J Nepal Med Assoc.

[13] Majstorovic M, Morse DE, Do D, Lim L, Herman NG, Moursi AM (2014). Indicators of dental anxiety in children just prior to treatment. J Clin Pediatr Dent.

[14] El-Housseiny AA, Alamoudi NM, Farsi NM, Derwi DA (2014). Characteristics of dental fear among Arabic-speaking children: a descriptive study. BMC Oral Health.

[15] El-Housseiny AA, Alsadat FA, Alamoudi NM, Derwi DA, Farsi DM, Attar MH, Andijani BM (2016). Reliability and validity of the children’s fear survey schedule-dental subscale for Arabic-speaking children: a cross-sectional study. BMC Oral Health.

[16] R Core Team (2022). R: A language and environment for statistical computing. R Foundation for Statistical Computing, Vienna, Austria. URL https://www.R-project.org/.

[17] ten Berge M, Veerkamp JS, Hoogstraten J, Prins PJ (2002). Childhood dental fear in the Netherlands: prevalence and normative data. Commun Dent Oral Epidemiol.

[18] Umamaheshwari N, Asokan S, Kumaran TS (2013). Child friendly colors in a pediatric dental practice. J Indian Soc Pedod Prev Dent.

[19] Centers for disease control and prevention COVID-19 Elastomeric respirators: strategies during conventional and surge demand situations conventional, contingency, and crisis strategies updated Oct. 13, 2020 https://www.cdc.gov/coronavirus/2019-ncov/hcp/elastomeric-respirators-strategy/index.html#print. Accessed 10 May 2022.

[20] Rank RCIC, Vilela JER, Rank MS, Ogawa WN, Imparato JCP (2019). Effect of awards after dental care in children's motivation. Eur Arch Paediatr Dent.

[21] Mendoza-Mendoza A, Perea MB, Yañez-Vico RM, Iglesias-Linares A (2015). Dental fear in children: the role of previous negative dental experiences. Clin Oral Investig.

[22] Alshoraim MA, El-Housseiny AA, Farsi NM, Felemban OM, Alamoudi NM, Alandejani AA (2018). Effects of child characteristics and dental history on dental fear: cross-sectional study. BMC Oral Health.

[23] Rojas-Alcayaga G, Uribe L, Barahona P, Lipari A, Molina Y, Herrera A, Ríos M (2015). Dental experience, anxiety, and oral health in low-income Chilean children. J Dent Child (Chic).

